# Supplementary figures and images for: Hierarchical Action of Mulberry miR156 in the Vegetative Phase Transition
Source: Int J Mol Sci. 2021 May 24;22(11):5550. doi: 10.3390/ijms22115550 (PMC8197408; doi:10.3390/ijms22115550)

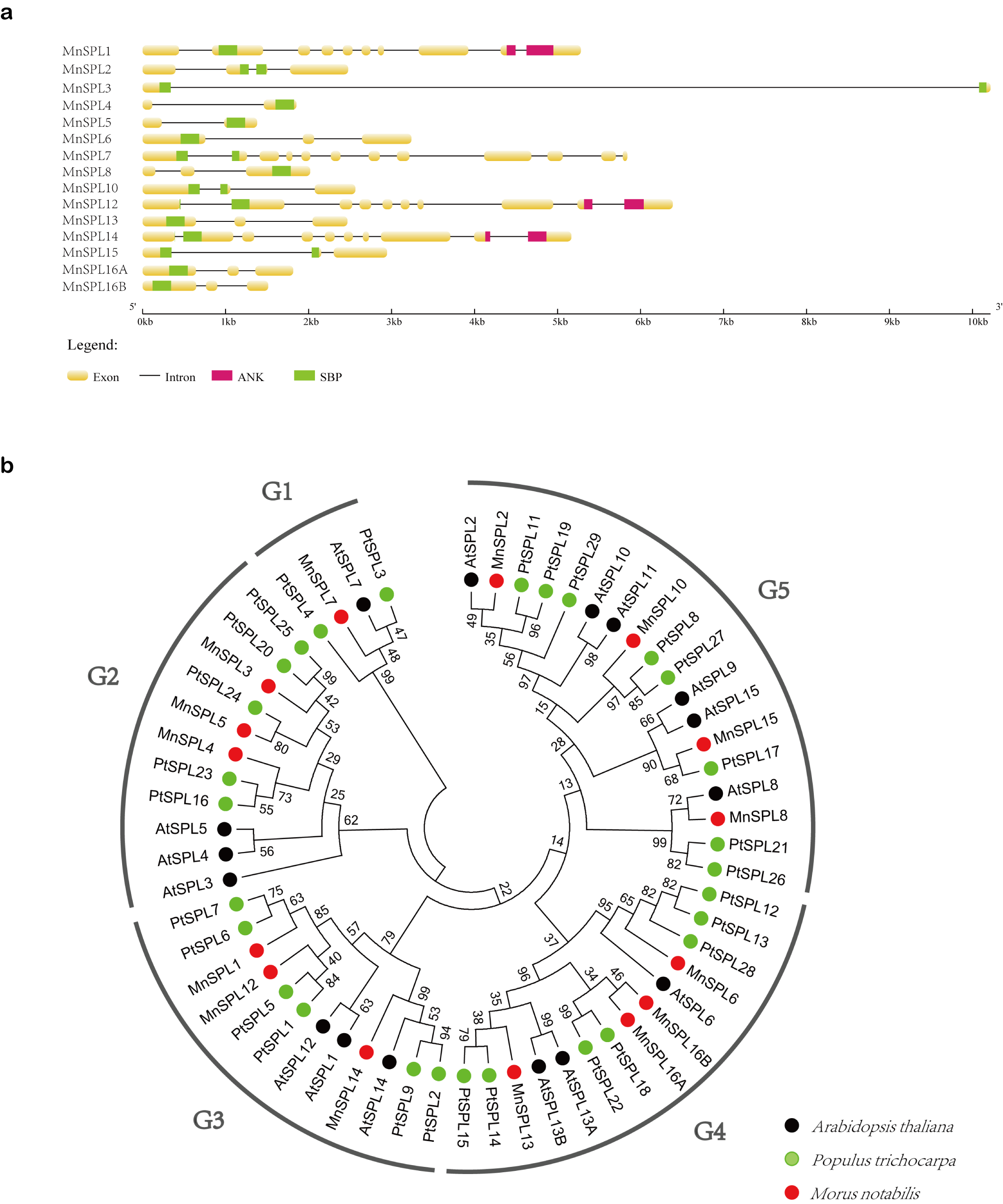

Supplement: Supplementary file 1 [file ijms-22-05550-s001.zip › ijms-1166087-supplmentary/Figure S1. SPL genes in mulberry. (a) Structural diagram of MnSPL genes. Yellow boxes show exons. Black lines show introns. Red and green boxes each represent ANK and SBP domains, respectively. (b) Neighb.tif]

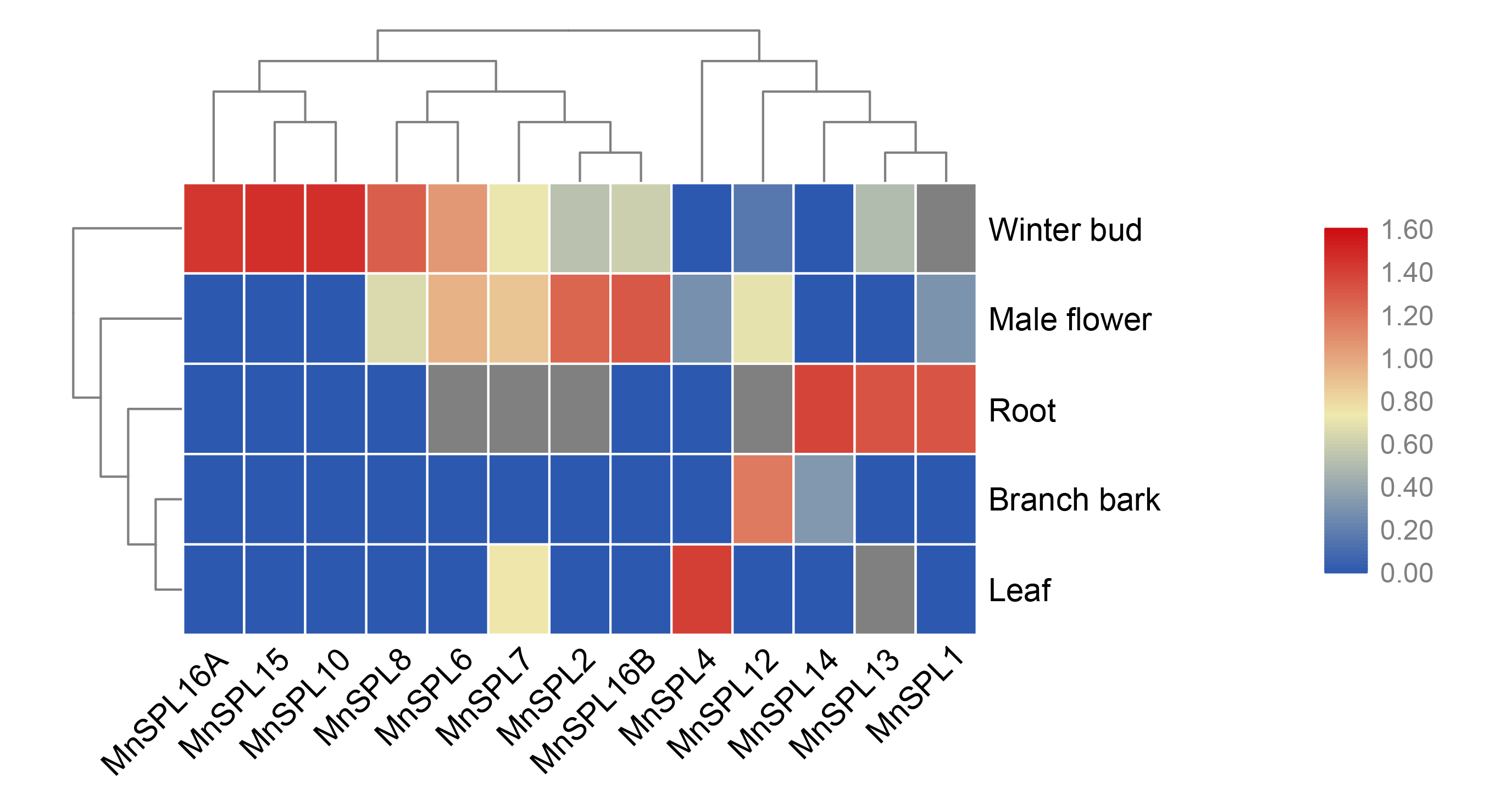

Supplement: Supplementary file 1 [file ijms-22-05550-s001.zip › ijms-1166087-supplmentary/Figure S2. Heat map showing the expression profile of SPL genes in five tissues from mulberry. Heat map was finished using TBtools, and the min-max normalization method was utilized to normalize the data..tif]
